# Locoregional delivery of chimeric antigen receptor-T cells: Breaking the spell in glioblastoma?

**DOI:** 10.1093/neuonc/noae063

**Published:** 2024-04-09

**Authors:** Marjolein Geurts, Matthias Preusser

**Affiliations:** The Brain Tumour Center at the Erasmus MC Cancer Institute, University Medical Center Rotterdam, Rotterdam, The Netherlands; Division of Oncology, Department of Medicine 1, Medical University, Vienna, Austria

Glioblastoma has become the prime example of a tumor that is resistant to immunotherapy. Randomized trials with immunotherapeutic strategies for glioblastoma have until now uniformly pointed towards a lack of therapeutic effectiveness.^1,2^ Several challenges may be responsible for this lack of effect. Glioblastomas are typified by their low immunogenic profiles, location in the T-cell sparse central nervous system, and are situated within a particularly immunosuppressive microenvironment.^3^ There is a critical need for the innovation of immunotherapeutic strategies that can effectively navigate and counter these challenges.^4^ A series of 3 articles published within few days, 2 in *Nature Medicine*^5,6^ and one in the *New England Journal of Medicine*,^7^ now highlight that locoregional application of chimeric antigen receptor (CAR)-T cells may be an approach for effective delivery of immunotherapy in high-grade glioma (Table 1). All 3 phase I studies explore the clinical tolerability and preliminary efficacy of innovative CAR-T cell constructs developed based on prior preclinical work.^8–10^

**Table 1.** Overview of 3 Recent Studies on Locoregional Delivery of Chimeric Antigen Receptor-T Cell Therapy of High-Grade Glioma

	Choi et al.^7^	Bagley et al.^6^	Brown et al.^5^
Study phase	1	1	1
Number of patients	3	6	65
Diagnosis (WHO 2021 classification)	Glioblastoma, *IDH* wild type	Glioblastoma, *IDH* wild type	Progressive/recurrent grade 3 or 4 malignant glioma
Molecular entry criterion (detection method)	EGFRvIII mutation detected (not specified)	Wild-type EGFR amplification detected (FISH)	IL-13Rα2 positive (IHC)
CAR-T cell construct	CARv3-TEAM-E	CART-EGFR-IL13Rα2	IL-13Rα2-CAR-T (Tcm), IL-13Rα2-CAR-T (Tn/mem)
Treatment target(s)	EGFR, EGFRvIII	EGFR, IL13Rα2	IL-13Rα2
Mode of administration	Invtraventricular (Ommaya reservoir)	Intrathecal (intraventricular reservoir)	Intratumoral, intraventricular, intratumoral/intraventricular
Dose-limiting toxicities	0	1	0
Radiographic responses	2 partial remissions, 1 near complete remission per RANO	6 (100%) with radiographic regression, none fulfilling objective response criteria per RANO	29/58 (50%) SD or better, 2 PR, 2 CR per modified RANO criteria

Brown et al. report on local delivery of interleukin-13 receptor alpha 2 (IL-13Rα2) targeted CAR-T cell therapy in high-grade glioma.^5^ IL-13Rα2 is a cancer-testis antigen that is expressed by the majority of malignant gliomas and is associated with a mesenchymal gene signature and poor prognosis.^8^ Antitumor immunity and complete tumor regression had previously been observed in one patient with recurrent multifocal glioblastoma after receiving both intraventricular and intratumoral administration of IL-13Rα2-targeted CAR-T cells.^11^ In the single-center, non-randomized, dose-escalation phase I study, 65 patients with first, second, third, or later recurrence of high-grade glioma (including 41 glioblastomas, isocitrate dehydrogenase gene, *IDH*, wild type, and a variety of grade 3 and 4 gliomas including cases with confirmed *IDH* mutation) with immunohistochemically confirmed tumoral IL-13Rα2 expression were treated with weekly infusions of IL-13Rα2-targeted CAR-T cell therapy intratumorally, intraventricularly or both intratumorally and intraventricularly. Two CAR-T cell manufacturing processes (Tcm and Tn/mem) were utilized, ultimately resulting in a 5-arm study. Primary objectives were feasibility and toxicity. Although no dose-limiting toxicities (DLT) were reported, one-third of the patients experienced grade 3 toxicity possibly or probably related to the CAR-T cells. Worth mentioning are one grade 3 encephalopathy and one grade 3 ataxia, as well as 2 patients with grade 4 cerebral edema shortly after cycle 1 of the CAR-T cells. Disease response rates and survival were secondary objectives, and evaluable in 58 patients. Stable disease or better was reached in 50% of these patients directly after the DLT period, including 2 partial and one complete response according to modified Response Assessment in Neuro-Oncology (RANO) criteria. Median overall survival of the 58 evaluable patients with mixed neuropathological diagnoses was 8 months (95% confidence interval 6.2, 10.1), while it was 7.7 months (95% confidence interval 6.0, 10.1) for the subset of patients with recurrent glioblastoma. Patients with recurrent glioblastoma treated with dual intratumoral/intraventricular application of the Tn/mem products on arm 5 exhibited longer survival as compared to patients treated with Tcm products on arms 1–4 (arm 5: 10.2 months; arms 1–4: 6.1 months; *P* = .02). However, although the investigators attempted to consider relevant covariates, these results need to be interpreted with caution due to the post hoc character of the analysis and the limited and heterogenous patient sample. Of note, patient-reported quality of life scores showed a modest, but significant, increase in slopes for arm 5 over arms 1–4 for recurrent glioblastoma participants (*P* = .027).

While it may be tempting to draw conclusions regarding the impact of locoregional delivery of IL-13Rα2-targeted CAR-T cells on survival, it is important to note that the study was neither designed nor powered to allow any claims on clinical benefit. Although half of the patients in this study demonstrated radiologically stable disease, the overall survival rates were not superior to those observed in clinical studies of recurrent glioma.^12–14^ Notably, the 3 patients who exhibited partial or complete responses all had *IDH*-mutated tumors. Furthermore, some patients received additional cycles of CAR-T cells outside the study protocol.

The exploratory translational endpoints demonstrate that CAR-T cells delivered intratumorally or intraventricularly were not only detectable in the cerebrospinal fluid (CSF) and tumor cavity fluid for > 7 days in a subset of patients, but were also able to traffic to the peripheral blood. Inflammatory cytokines, particularly those associated with the interferon γ (IFNγ) pathway, were notably elevated in the CSF post-treatment, suggesting their potential as biomarkers for assessing CAR-T cell activity. The levels of CD3-positive T cells in the tumor before treatment were linked to patient survival in this small study; however, it remains uncertain whether this is an indication of a potential prognostic or predictive role for this biomarker. In conclusion, this study demonstrates the feasibility of locoregional IL-13Rα2-targeted CAR-T cell therapy in high-grade glioma, offering significant translational insights crucial for advancing CAR-T cell therapies in glioma treatment. For further development, the investigators identified arm 5 procedures as the most promising among the investigated approaches.

Bagley et al. report the results of non-prespecified interim analysis of 6 patients treated in the first 2 dose levels of an ongoing first-in-human phase 1 study with a bivalent CAR-T cell product simultaneously targeting epidermal growth factor receptor (EGFR) and IL13Rα2.^6^ Patients with an *IDH* wild-type glioblastoma that had recurred after prior radiotherapy and presence of *EGFR* amplification by fluorescence in situ hybridization on any prior tumor tissue specimen could be included. Presence of IL13Rα2 was not required as a study inclusion criterion. All patients underwent maximal safe resection of the recurrent tumor to confirm the *EGFR* amplification as target, and an intraventricular subcutaneous reservoir (Ommaya) was placed in the same procedure. The cell product was subsequently injected intrathecally as a single dose through the reservoir 17–35 days after surgery.

The primary objective of the study by Bagley et al. is to evaluate the safety of CART-EGFR-IL13Rα2 cells in patients with recurrent glioblastoma. All 6 patients reported now experienced early and moderate-severe neurotoxicity with elements of both immune effector cell-associated neurotoxicity syndrome (ICANS) and tumor inflammation-associated neurotoxicity, which was managed with high-dose dexamethasone and the interleukin-1-receptor antagonist anakinra. One patient in dose level 2 experienced a DLT (grade 3 anorexia, generalized muscle weakness, and fatigue). Reductions in the size of the enhancing tumor were observed in all 6 patients on the first magnetic resonance imaging (MRI) scan obtained 24–48 hours after CAR-T cell administration, with partial tumor regression maintained at day + 28 and beyond in a subset of cases. However, none met criteria for an objective response per modified RANO criteria. Patients were treated between June 2023 and January 2024, resulting in short follow-up time for most patients. During follow-up, 5 out of 6 patients experienced increased contrast enhancement on MRI shortly after treatment. In one patient, this enhancement was resected, revealing therapy-related changes and rare atypical glial cells comprising approximately 10% of the total cellularity. Two patients received bevacizumab, and one received dexamethasone for symptom relief. Contrast enhancement decreased in all patients over time. CAR-T-EGFR-IL13Rα2 cells were detected in peripheral blood in all patients, indicating communication between the CSF and peripheral blood compartments. CSF cytokine levels supported evidence of CAR-T cell activation and cytotoxic activity, with IFNγ, interleukin 2 (IL-2), tumor necrosis factor α (TNFα), and IL-6 showing rapid increases in CSF before returning to baseline levels within 2 weeks, consistent with preclinical data.

To conclude, the findings of Bagley et al. demonstrate the safety of intraventricular administration of a bivalent CAR-T cell product targeting both EGFR and IL13Rα2 along with promising tumor responses (albeit not meeting objective response criteria per modified RANO criteria). The durability of these responses and their impact on overall survival are eagerly anticipated.

Choi et al. report data from a prespecified interim analysis of the first 3 patients with *IDH* wild-type recurrent glioblastoma treated in the first-in-human, investigator-initiated, open-label INCIPIENT study.^7^ The innovative CAR-T cell construct CARv3-TEAM-E applied in this study targets EGFRvIII antigen, as well as the wild-type EGFR protein via secretion of a T-cell–engaging antibody molecule (TEAM). In all 3 patients treated in the INCIPIENT trial so far, the tumor tissue from the initial resection in the newly diagnosed setting was EGFR variant III tumor-specific (EGFRvIII) positive, and in 2 of these 3 patients also the tumor tissue resected at progression prior to the CAR-T cell therapy was EGFRvIII positive. In one patient (participant 3), however, tissue analysis at the time of surgery for recurrence revealed a loss of EGFRvIII expression. Two patients received a single infusion of 10 × 10^6^ CAR-positive CARv3-TEAM-E T cells and one patient 2 intraventricular infusions (37 days apart) through an Ommaya reservoir. Grade 3 events probably related to the investigational product included grade 3 encephalopathy for 3 days in one patient and grade 3 fatigue for 8 days in another patient. All 3 patients experienced fevers peaking on day 2 and managed with anakinra. No associated DLT were described. CAR-T cells could transiently be detected in the CSF and the peripheral blood. Of particular interest are impressive radiographic responses reported in all 3 patients, although they were only transient in 2 cases. In participant 1, rapid regression of the tumor was already seen on day 1 after the CAR-T cell infusion and disease progression occurred after 2 weeks. In participant 2, a decrease in cross-sectional tumor area by 18.5% on day 2 and by 60.7% on day 69 with maintained disease stability for more than 150 days was reported. Participant 3 showed near complete regression in an MRI scan obtained 5 days after CAR-T cell infusion and recurrence was detected within one month. Overall, the interim results of the ongoing INCIPIENT study provide proof of principle for CAR-T cell targeting of multiple tumor cell-associated surface antigens, substantiate EGFR as treatment target of interest, provide initial safety data for intraventricular application and indicate potential antitumor efficacy for the CARv3-TEAM-E construct. Going forward it will be important to understand mechanisms of resistance and establish optimal application regimens, potentially also exploring combination strategies with conditioning chemotherapy. Furthermore, the predictive value of the EGFRvIII biomarker used for patient selection in the INCIPIENT study will need to be clarified in further studies.

Overall, the 3 current studies show that locoregional administration of CAR-T cells in glioma is safe, biologically active, and associated with remarkable radiographic responses with quick and pronounced regression of contrast-enhancing tumor portions on MRI in individual cases. Although systematic comparisons of the toxicity profiles of locoregional versus systemic CAR-T cell delivery are missing so far, locoregional delivery may be helpful in limiting systemic adverse events. Of note, however, CAR-T cells were transiently found in the peripheral blood of some patients and may contribute to toxicities also outside of the CNS even after locoregional application. In 2 of the 3 studies, objective response criteria per internationally accepted RANO response criteria were fulfilled, in some cases even within few days. At present, however, it is unclear what exactly these radiological changes signify, as further insights, for example, from molecular imaging or systematic histopathological evaluations obtained before and after application of locoregional CAR-T cells are lacking so far. Furthermore, the radiographic regressions were transient in most cases and follow-up times and patient numbers are too limited to evaluate long-term disease evolution after this experimental treatment. Still, CAR-T cell therapy is emerging as a promising immunotherapeutic approach for high-grade glioma, and there is eager anticipation for the longer-term survival outcomes from these studies. At present, however, this treatment must be viewed as purely experimental and should be restricted to application within dedicated clinical studies. Ongoing clinical trial activities of CAR T cells in glioma include different CAR-T targets like B7-H3 (NCT04077866; NCT05241392; NCT04385173), matrix metallopeptidase 2 (MMP2; NCT05627323; NCT04214392), CD70 (NCT05353530), as well as EGFRvIII (NCT06186401).^15^ Additionally, there is a study combining IL-13Rα2-targeted CAR-T cells with checkpoint inhibitors (NCT04003649). These endeavors promise to yield additional insights into safety and efficacy, thus paving the way for future advancements in the field. The next steps will involve assessing clinical efficacy further to confirm the promising signals observed so far and to eventually determine the optimal implementation of this novel approach into clinical practice.
